# Electrochemical Sensing of α-Fetoprotein Based on Molecularly Imprinted Polymerized Ionic Liquid Film on a Gold Nanoparticle Modified Electrode Surface

**DOI:** 10.3390/s19143218

**Published:** 2019-07-22

**Authors:** Yingying Wu, Yanying Wang, Xing Wang, Chen Wang, Chunya Li, Zhengguo Wang

**Affiliations:** 1 Institute of Food Science and Engineering Technology, Hezhou University, Hezhou 542899, China; 2 Key Laboratory of Analytical Chemistry of the State Ethnic Affairs Commission, College of Chemistry and Materials Science, South-Central University for Nationalities, Wuhan 430074, China

**Keywords:** molecularly imprinted, α-fetoprotein, ionic liquid, gold nanoparticles

## Abstract

A molecularly imprinted sensor was fabricated for alpha-fetoprotein (AFP) using an ionic liquid as a functional monomer. Ionic liquid possesses many excellent characteristics which can improve the sensing performances of the imprinted electrochemical sensor. To demonstrate this purpose, 1-[3-(N-cystamine)propyl]-3-vinylimidazolium tetrafluoroborate ionic liquid [(Cys)VIMBF_4_] was synthesized and used as a functional monomer to fabricate an AFP imprinted polymerized ionic liquid film on a gold nanoparticle modified glassy carbon electrode (GCE) surface at room temperature. After removing the AFP template, a molecularly imprinted electrochemical sensor was successfully prepared. The molecularly imprinted sensor exhibits excellent selectivity towards AFP, and can be used for sensitive determination of AFP. Under the optimized conditions, the imprinted sensor shows a good linear response to AFP in the concentration range of 0.03 ng mL^−1^~5 ng mL^−1^. The detection limit is estimated to be 2 pg mL^−1^.

## 1. Introduction

Alpha-fetoprotein (AFP) with a molecular weight of approximately 70 kDa is always considered as a vital and specific biomarker for diagnosing, treating, and the prognosis of liver cancer. In healthy human serum, the concentration of AFP is often lower than 20 ng mL^−1^. However, when suffering from liver cancer, the AFP concentration in serum will increase significantly [1]. Thus, the AFP level can provide a good standard for the early diagnosis of liver cancer, and will benefit for early treatment and help to prolong survival and improve the quality of life. Hence, to determine AFP concentration in human serum is extremely important for patients who suffer from liver cancer. To date, for measuring AFP with high performances, many immunoassay methods based on fluorescence spectroscopy [2,3], chemiluminescence [4,5], electrochemiluminescence [6,7], enzyme-linked immunosorbent assay [8], surface plasmon resonance [9], electrochemical immunosensing [10,11] and photoelectrochemical sensing [12,13] have been successfully developed. Although some of these methods can give good sensitivity and selectivity for meeting the requirements for AFP determination, they still need to overcome many obstacles, such as time-consuming or sophisticated instruments, etc. In addition, in the immunoassay process, an antibody is indispensable for improving the selectivity of the sensing system. Nevertheless, when using an antibody as a recognition element, the operation conditions such as temperature and pH value of the solution should be controlled strictly as the antibody is a natural biomolecule which can lose its bioactivity under harsh conditions. Moreover, the strong affinity between the antibody and antigen is essential to the disadvantage of the reusability of the immunosensors, thus, the practical applications of immunosensing systems are restricted. Therefore, to seek recognition elements other than antibody for sensing biomarkers with high sensitivity and selectivity is still a challenge for electrochemical biosensor fabrication.

The molecularly imprinted technique is a promising procedure for producing artificial receptors in polymer substrates. Template molecules and functional monomers are thoroughly pre-assembled through the covalent bond or non-covalent bond. Then, a polymerization reaction is initialized to form a polymer network in which all template molecules are embedded with specific spatial structure and interactions. The template molecules can be removed from the polymer by just simply being washed with proper solvent to produce some imprinted sites. The imprinted sites are very complementary to the templates in structure, spatial configuration and chemical interaction, thus show excellent selectivity and can be used as a recognition element for sensing system. Compared to the natural recognition elements, molecularly imprinted polymer noted as an artificial antibody provides some advantages such as low cost, good stability, easy fabrication and potential application in harsh conditions. Up to now, the molecularly imprinted technique has been extensively used in many fields such as extraction [14], separation [15], catalysis [16], fluorescent sensing [17], electrochemical sensing [18] and photoelectrochemical sensing [19], etc. In the fabrication of a sensing platform for a small molecule, molecularly imprinted polymer shows significant progress [20]. However, in the case of the biomacromolecular imprinting polymer fabrication, challenges still exist due to the fact that the imprinted procedure for small molecules is not suitable for biomolecules. An aqueous solution is desirable for biomolecular imprinted polymer fabrication, but small molecule imprinting is often conducted in the organic phase. An aqueous solution is desirable for biomolecular imprinting polymer fabrication, nevertheless, small molecule imprinting is often conducted in the organic phase. Thus, to exploit functional monomer which can be polymerized in the aqueous phase for biomolecules is very important. Some electrochemical sensors for AFP based on molecularly imprinted polymer have been reported recently. Using AFP as a template, molecularly imprinted sensing platforms have been fabricated and demonstrated efficient for AFP determination. Based on a boronate affinity molecular imprinting technique and functionalized SiO_2_@CQDs/AuNPs/MPBA nanocomposites, a boronate-affinity sandwich electrochemiluminescence sensor was constructed for AFP [21]. By using AFP as template and dopamine as functional monomer, an AFP imprinted immunosensor based on a glassy carbon electrode modified with polythionine and gold nanoparticles was fabricated [22]. On a Chitosan GCE surface, using AFP as a template, acrylamide as functional monomer and *N*,*N*-methylene bisacrylamide as crossing linker, a molecularly imprinted sensor was prepared for AFP [23]. All these imprinted sensors show high sensitivity and selectivity.

Ionic liquid is demonstrated to be a suitable functional monomer for fabricating molecularly imprinted polymer for biomolecules due to its intrinsic characteristics [24,25]. Ionic liquid is a sort of organic salt which usually comprises of large organic cation and small inorganic or organic anions. They possess good conductivity, stability, low melting point, and some of them can be dissolved in water by adjusting their chemical structure. Compared with acrylic polymers and conducting polymers, ionic liquids possess excellent electrical conductivity. In addition, the functionality of ionic liquids can be designed according to the requirements of the experiment. Thus, ionic liquids are good candidates for fabricating sensing platforms, especially for electro- and photoelectrochemical biosensors. Herein, N-Cystamine functionalized ionic liquid was synthesized and used as a functional monomer for developing a molecularly imprinted sensor for AFP. The molecularly imprinted procedure was carried out in an aqueous solution containing the AFP template, ionic liquid functional monomer and initiator. The polymerization was initialized under room temperature to maintain the bioactivity, spatial configuration and chemical structure of AFP, and thus to improve the selectivity and sensitivity of the imprinted sensor. Experimental conditions were optimized for the investigation of the performances of the imprinted sensor. A novel method for AFP determination was developed successfully.

## 2. Experimental

### 2.1. Materials and Reagents

1-Vinylimidazole, cystamine, di-tert-butyl dicarbonate ester, 1,3-dibromopropane, chloroauric acid, ascorbic acid (AA), L-cysteine (L-Cys), glycine (Gly), L-histidine (L-His), and sodium perchlorate were purchased from Sinopharm Chemical Reagent Co., Ltd. (Shanghai, China). AFP was brought from Shanghai Linc-Bio Science Co. LTD (Shanghai, China). Human serum albumin (HSA), neuron special enolase (NSE), prostatic specific antigen (PSA), and immunoglobulin G (IgG) were purchased from Beijing Bioss Biotechnology Co. LTD (Beijing, China). Other reagents were analytical grade and were used without purifying. All the aqueous solutions were prepared by using ultrapure water.

Electrochemical measurements were performed with CHI 660 C electrochemical workstation (Chenhua Corp., Shanghai, China). A conventional three-electrode system was employed with a glassy carbon working electrode in diameter of 3 mm (GCE, Wuhan Gaossunion Technology Co., Ltd., Wuhan, China), a Pt wire auxiliary electrode and a saturated calomel reference electrode. Scanning electron microscope (SEM) was carried out with SU 8010 scanning electron microscope (Hitachi, Japan). The mass spectrum was obtained on Q-TOF-MS 6520A HPLC-MS spectrograph (Agilent, Palo Alto, CA, USA). ^1^H-NMR spectra were supplied by AVANCE III 400 nuclear magnetic resonance spectrometer (Bruker, Karlsruhe, Baden-Württemberg, France). UV/Vis analysis was performed on PE Lambda Bio 35 (PerkinElmer, Waltham, MA, USA). FTIR spectra were conducted on Nicolet NEXUS-470 FTIR spectrometer. All reagents and apparatus are detailed in the Supporting Information.

### 2.2. (Cys)VIMBF_4_ Ionic Liquid Synthesis

(Cys)VIMBF_4_ ionic liquid was synthesized according to the previously reported procedure with some modifications [26] and was briefly depicted as the following:

1,3-dibromopropane (20.10 g, 0.1 mol) was dissolved in 50 mL anhydrous acetonitrile and was loaded into a 150 mL three-necked flask. After being purged with nitrogen for 30 min, N-ethylene imidazole (0.82 g, 0.01 mol) was added. The mixture was heated to 60 °C and kept reacting for 12 h. Solvent was removed by vacuum distillation. The resulted product was thoroughly washed with ether to give 1-bromopropyl-3-vinylimidazolium bromine (Product A).

Cystamine (4.50 g, 20.0 mmol) and triethylamine (8.36 mL, 60.0 mmol) were dissolved in 60 mL methanol. After being cooled to 0 °C, di-tert-butyl dicarbonate ester (2.182 g, 10.0 mL) in 40 mL methanol was drop added into the mixed solution. Then, the mixture was kept reacting for 6 h. The solvent was removed by vacuum distillation to give a creamy yellow solid product. The product was dissolved in NaH_2_PO_4_ solution (1 mol L^−1^) and the pH value was adjusted to 9.0. Subsequently, ethyl acetate was used to extract the ionic liquid from the aqueous solution, and was dried by anhydrous MgSO_4_. Ethyl acetate was removed and the obtained yellow oily product was dried under vacuum to give the target ionic liquid (product B).

**Scheme 1:**
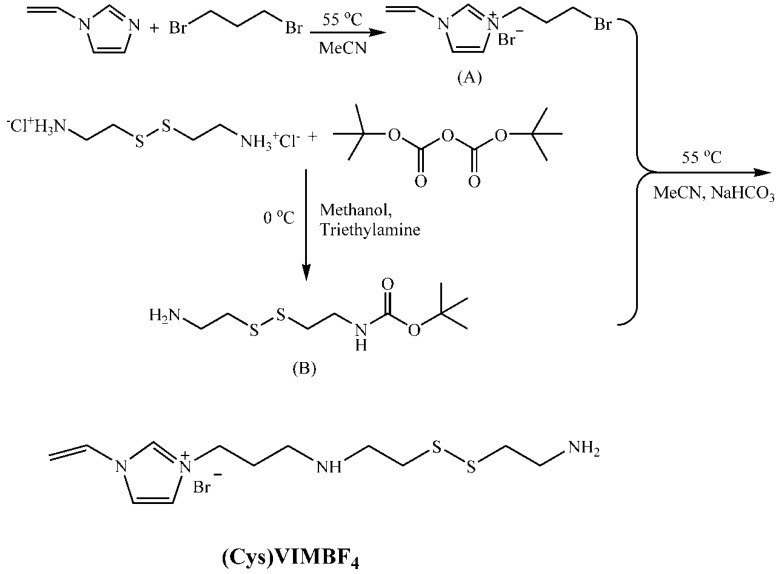
Schematics for the synthetic route of (Cys)VIMBF_4_ ionic liquid.

Product (A) (1.6 g, 5.6 mmol) was dissolved in anhydrous acetonitrile (20 mL), and stirred for 0.5 h under the protection of nitrogen. Then, 20 mL of acetonitrile containing (2.1 g, 8.3 mmol) product (B) was added slowly. The mixture was heated to 55 °C and kept reacting for 24 h. After removing the solvent with vacuum distillation, the mixture was extracted by water and dichloromethane. The aqueous solution was collected and distilled under vacuum to produce yellow oily liquid. The product was dissolved in dichloromethane, slowly adding HCl (4 mol/L)/dioxane and dichloromethane, and stirred at room temperature for 24 h to obtain yellow viscous solid. The solid was washed several times with dichloromethane, then dissolved in methanol, regulated to weak alkaline with NaHCO_3_, vacuum dried to obtain the target product (yield 1.6 g, 76.3%). Schematic for the synthesis of the (Cys)VIMBF_4_ ionic liquid was presented in Scheme 1. ^1^H-NMR and ^13^C-NMR were used to characterize (Cys)VIMBF_4_ ionic liquid. All results were shown in Figures S1 and S2.

### 2.3. Gold Nanoparticles Modified Glassy Carbon Electrode Preparation.

A glassy carbon electrode was polished using aluminum oxide slurry, and then was cleared with ultrasonication in nitric acid (v/v, 1:1), ethanol and ultrapure water. After being dried under ambient temperature, the procedure for electrochemical deposition of gold nanoparticles on the GCE surface was carried out in a sodium perchlorate solution containing 0.1 mM chloroauric acid. Chronoamperometry was used to initialize the deposition with a potential step from 0.89 V to −0.8 V. The pulse width of 10 s was controlled. Subsequently, the electrodeposition was performed by using cyclic voltammetry. The parameters were set as the following: initial potential −0.04 V, potential range from 0.3 V to −0.04 V, scan rate 50 mV/s and 60 cycles. Finally, the gold nanoparticle modified electrode was thoroughly rinsed with ultrapure water to give a nanointerface, e.g., AuNPs/GCE. Gold nanoparticles not only possess excellent conductivity, but also possess a large specific surface area, which can help to increase the loading amount of ionic liquid monomer and protein, thus helping to improve the efficiency of the imprinting and enhancing the sensitivity of the imprinted sensor.

### 2.4. AFP Molecularly Imprinted Sensor Fabrication

**Scheme 2:**
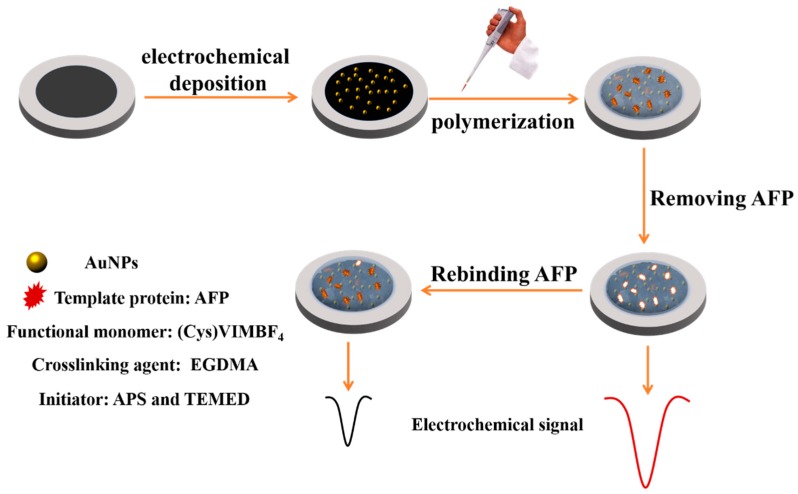
Schematic illustration for the alpha-fetoprotein (AFP) imprinted sensor fabrication and the electrochemical responses.

AuNPs/GCE was incubated into a (Cys)VIMBF_4_ solution at the concentration of 0.01 M for 12 h at the temperature of 25 °C to accomplish a self-assembling process. Then, the AuNPs/GCE was thoroughly washed with ultrapure water and dried at room temperature. Subsequently, AFP (100 ng mL^−1^) in Tris-HCl solution (pH 7.4), (Cys)VIMBF_4_ ionic liquid solution and Ethyleneglycol Dimethacrylate(EGDMA) solution was thoroughly mixed in a centrifugal tube. After adding Ammonium Persulphate (APS) and N,N,N′,N′-Tetramethylethylenediamine(TEMED), 5 µL of the mixture solution was coated onto the aforementioned AuNPs/GCE surface and was initialized to form a polymerized ionic liquid film electrode. After that, the electrode was successively washed with 10% (v/v) HAc-10% (w/w) SDS solution, Tris-HCl (0.1 mol L^−1^, pH 7.4) solution and ultrapure water to remove AFP template to form a molecularly imprinted sensor which can be denoted as MIP-poly[(Cys)VIMBF_4_]/AuNPs/GCE. A non-imprinted sensor was fabricated with a similar procedure just by substituting AFP solution with an equal volume of Tris-HCl solution. The non-imprinted sensor was denoted as NIP-poly[(Cys)VIMBF_4_]/AuNPs/GCE. The prepared imprinted sensor and non-imprinted sensor were stored at the temperature of ~4 °C for further investigations. The schematics for the sensor fabrication and working process of AFP were depicted in Scheme 2.

### 2.5. Electrochemical Measurements

All electrochemical experiments were carried out in a 10 mL electrolytic cell with a three-electrode system using the imprinted or non-imprinted film electrode as a working electrode, a platinum wire electrode as a counter electrode, and a saturated calomel electrode as a reference electrode. For the cyclic voltammetric and electrochemical impedance characterizations, K_3_[Fe(CN)_6_]/K_4_[Fe(CN)_6_] at the concentration of 5.0 mM was used as redox probes. 0.01 M phosphate buffer was used as the electrolytic solution. In the procedure for AFP determination, the imprinted sensor was incubated into the AFP solution for 12 min to selectively rebind to AFP antigen which was immobilized on the interface surface, the peak current variation before and after interacting with AFP was used as the signal to develop a method for AFP determining.

## 3. Results and Discussion

### 3.1. Characterizations

**Figure 1:**
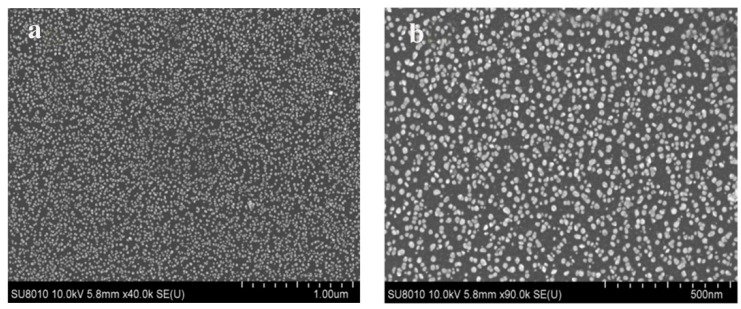
Scanning electron microscopic images of gold nanoparticles modified glassy carbon electrode surface.

Scanning electron microscopy was used to characterize the morphology of the gold nanoparticles modified glassy carbon electrode surface. As shown in Figure 1, nanoparticles were distributed on the electrode surface homogeneously (a). After being magnified (b), we can see gold nanoparticles with irregular shape have been electrodeposited onto the glassy carbon electrode surface successfully. These gold nanoparticles can supply a high specific surface area and some active-sites for immobilizing functional monomer, and thus promoting the sensitivity and selectivity of the imprinted sensor.

**Figure 2:**
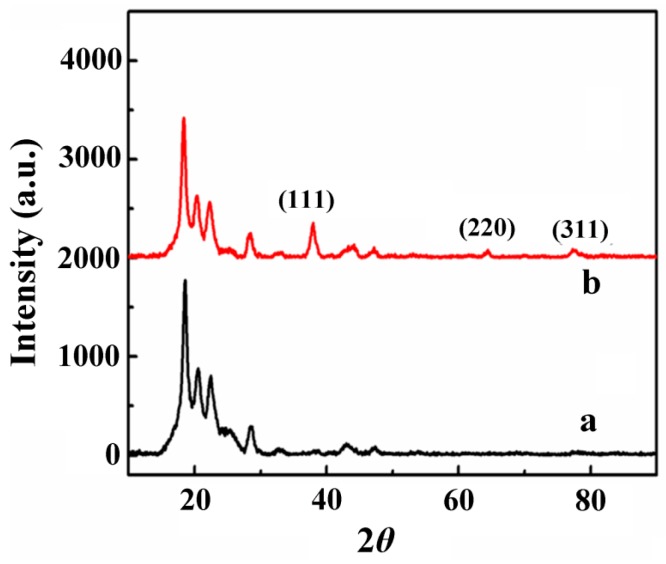
A glassy carbon electrode before (**a**) and after (**b**) being modified with gold nanoparticles was characterized with X-ray diffraction.

Before and after gold nanoparticles were electrochemically deposited, the glassy carbon electrode surface was characterized by X-ray diffraction. As shown in Figure 2, a significant difference can be observed for the diffraction peaks of a glassy carbon electrode before (a) and after (b) being modified with gold nanoparticles. As gold nanoparticles were electrochemically deposited onto the electrode surface, three diffraction peaks located at 38.252°, 64.693°, 81.882° appear obviously, and corresponding to the lattice planes of (111), (220) and (311) of a cubic crystal of gold, indicating the successful fabrication of the gold nanoparticles modified electrode.

**Figure 3:**
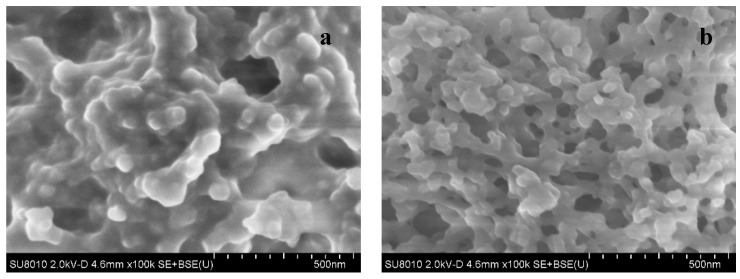
Scanning electron microscopic images of an imprinted film modified electrode before (**a**) and after (**b**) removing AFP.

The images of the imprinted film modified electrode surface before (a) and after (b) removing AFP template were characterized with scanning electron microscopy and were presented as Figure 3. From the comparison of these images, after AFP was removed, the morphology was significantly different from the film containing AFP, meaning the successfully fabricated the AFP imprinted sensing platform. The XPS characterizations of the AFP imprinted film before (a) and after (b) removing AFP, and the non-imprinted film (c) were presented in Figure S3. The content of S, C, N, O in the imprinted film before and after removing AFP, and the non-imprinted film was calculated and shown in Table S1. It is obvious to see that the ratio of O increases significantly after AFP was introduced into the polymerized ionic liquid film. This fact is mainly attributed to the oxygen content in the AFP is higher than that in the (Cys)VIMBF_4_ ionic liquid. Meanwhile, with AFP removed from the imprinted film, the oxygen content decreased accordingly, and was similar to that in the non-imprinted film, meaning the successfully removed AFP to produce imprinted sites.

**Figure 4:**
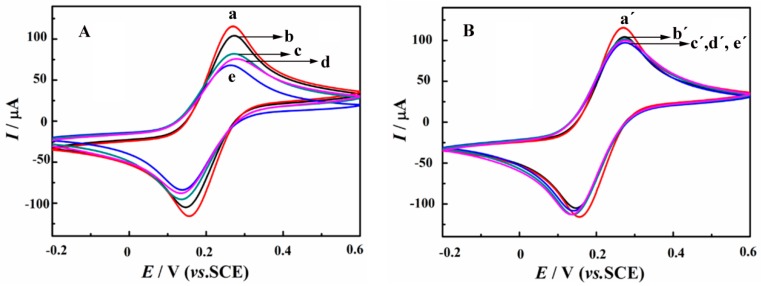
(**A**) Cyclic voltammograms of an AuNPs/GCE (**a**), a bare GCE (**b**), a MIP-poly[(Cys)VIMBF_4_]/AuNPs/GCE before (**e**) and after (**c**) removing AFP, and rebinding AFP (**d**); (**B**) Cyclic voltammograms of an AuNPs/GCE (**a**´), a bare GCE(**b**´), a NIP-poly[(Cys)VIMBF_4_]/AuNPs/GCE before (**e**´) and after (**c**´) removing AFP, and rebinding AFP (**d**´); AFP used for incubation is at the concentration of 20.0 ng mL^−1^; Scan rate: 0.1 V s^−1^.

Cyclic voltammetry was employed for the characterization of the imprinted (A) and the non-imprinted sensors during the stepwise fabrication process. Cyclic voltammograms were presented as Figure 4. As gold nanoparticles were electrochemically deposited on the unmodified glassy carbon electrode surface, the electrode surface was enlarged, thus leading to the peak current response of K_3_[Fe(CN)_6_]/K_4_[Fe(CN)_6_] which increased significantly (curve a). However, when the molecularly imprinted film was polymerized onto the AuNPs/GCE surface, the peak current response of K_3_[Fe(CN)_6_]/K_4_[Fe(CN)_6_] decreases dramatically due to the poor conductivity of AFP proteins which can retard the electron transfer and mass transfer (curve e). After removing AFP, the peak current response increases immediately due to the exposure of the imprinted sites again (curve c). Furthermore, AFP was encouraged to selectively rebind to the imprinted sites, leading to the retarded effects to electroactive probes. As a result, the peak current response decreases appropriately (curve d). In the case of the non-imprinted sensor, cyclic voltammetric behaviors of K_3_[Fe(CN)_6_]/K_4_[Fe(CN)_6_] at the unmodified glassy carbon electrode (curve b´) and AuNPs/GCE (curve a´) are similar to that for the imprinted sensor. Nevertheless, the cyclic voltammetric behaviors of K_3_[Fe(CN)_6_]/K_4_[Fe(CN)_6_] at the non-imprinted sensor before (curve e´) and after (curve c´) removing AFP, and rebinding AFP (curve d´) show significant differences to the imprinted sensor. The variations for the peak current among these three steps for the non-imprinted sensor fabrication are slight, meaning no imprinted sites have been produced in the absence of AFP template. Thus, the non-imprinted sensor will be not suitable for selectively determining AFP.

**Figure 5:**
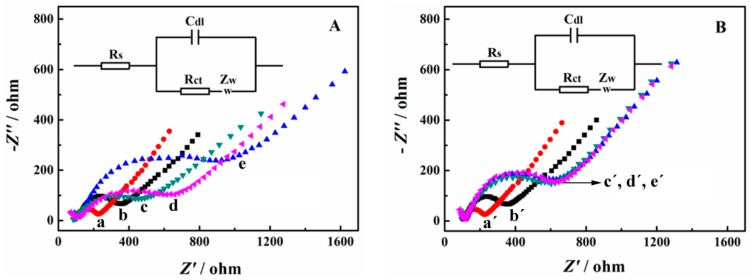
(**A**) Nyquist plots of an AuNPs/GCE (**a**), a bare GCE (**b**), a MIP-poly[(Cys)VIMBF_4_]/AuNPs/GCE before (**e**) and after (**c**) removing AFP, and rebinding AFP (**d**); (**B**) Nyquist plots of an AuNPs/GCE (**a´**), a bare GCE (**b´**), a NIP-poly[(Cys)VIMBF_4_]/AuNPs/GCE before (**e´**) and after (**c´**) washing, and interacting with AFP (**d´**) at the concentration of 20.0 ng mL^−1^; Supporting electrolyte: 5.0 mmol L^−1^ K_3_[Fe(CN)_6_]/K_4_[Fe(CN)_6_] (1:1) + 0.01 mol L^−1^ phosphate buffer (pH 7.4) + 0.1 mol L^−1^ KCl solution.

The fabrication procedures for the imprinted and the non-imprinted sensors were also characterized by electrochemical impedance spectroscopy. The Nyquist plots of K_3_[Fe(CN)_6_]/K_4_[Fe(CN)_6_] (5.0 mmol L^−1^) at each step for the fabrication of the imprinted (A) and the non-imprinted (B) sensor were presented in Figure 5. Electrochemical impedance spectroscopy is an efficient tool for characterization of the properties of these sensing interfaces. The Nyquist plot shows a semicircle in high frequency and straight line in low frequency, which is corresponding to a charge transfer control and diffusion control process respectively. The diameter of the semicircle can provide the information for evaluating the charge transfer resistance directly. As shown in Figure 5, when gold nanoparticles were modified on a glassy carbon electrode surface, the diameter of the semicircle decreased obviously, meaning gold nanoparticles can improve the electron transfer rate. After AFP imprinted polymerized ionic liquid film was integrated onto AuNPs/GCE surface, the charge transfer resistance increases dramatically due to the poor conductivity of AFP (curve e). As AFP was removed from the polymer film, the charge transfer resistance decreases immediately (curve c), indicating that some imprinted sites have been produced for selective recognition of AFP. Upon being incubated into AFP solution at the concentration of 10 ng mL^−1^ for 12 min, AFP can rebind to the imprinted sites due to the matching of the spatial structure and chemical interaction, and thus leading to the charge transfer resistance increases accordingly. On account of the charge transfer resistance whose variation is related to AFP concentration, the imprinted sensor will be suitable for AFP determination. On account of the charge transfer resistance, variation is related to AFP concentration, the imprinted sensor will be suitable for AFP determination. In the case of the non-imprinted sensor, Nyquist plots for K_3_[Fe(CN)_6_]/K_4_[Fe(CN)_6_] at the sensing interface before AFP removing (curve e´), after AFP removing (curve c´) and rebinding AFP (curve d´) are distinctly different from that of the imprinted sensor. No obvious variation was observed on the diameters of these semicircles, indicating the charge transfer resistance changes slightly, thus no imprinted sites were created in the process for the non-imprinted sensor fabrication. In order to verify the variation of the electrochemical properties of these interfaces, the equivalent circuit model inserted in Figure 5 was used to fit the experimental data using ZSimDemo. The *R*ct values for the AuNPs/GCE and the bare GCE were 99.9 Ω and 208.2 Ω, respectively. AFP imprinted polymer was introduced onto the AuNPs/GCE, and *R*ct increased to 906.2 Ω. After AFP removal, *R*ct decreased to 391.7 Ω. Meanwhile, with AFP rebinding to the interface, *R*ct increased to 542.1 Ω. Compared to the MIP sensor, *R*ct value on the NIP sensor surface was also simulated. *R*ct values on the NIP-poly[(Cys)VIMBF_4_]/AuNPs/GCE before (e´) and after (c´) removing AFP, and rebinding AFP (d´) were 485.9 Ω, 463.1 Ω and 478.9 Ω. The slight change in *R*ct value means no imprinted sites were produced on the polymerized ionic liquid film surface.

### 3.2. Optimizing Experimental Conditions

#### 3.2.1. Effect of the pH Value

**Figure 6:**
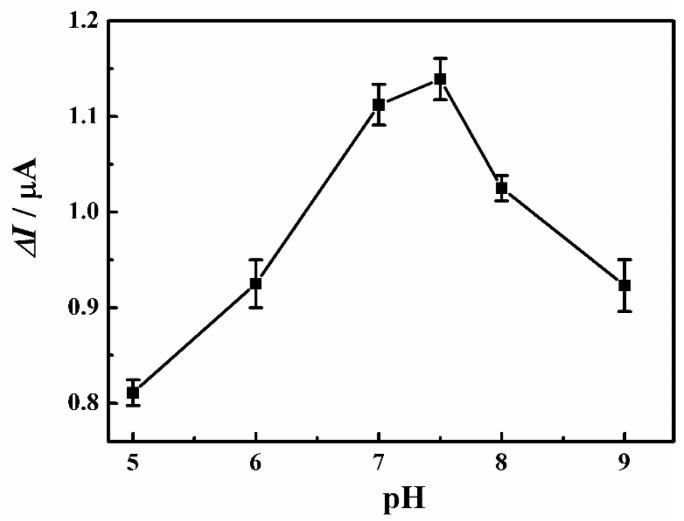
Influence of the pH value on the current response of the imprinted sensor towards AFP at the concentration of 1.0 ng mL^−1^.

The pH value of the AFP solution for incubation will influence the configuration and charge property of AFP protein, thus affecting the rebinding behavior of AFP to the imprinted sites. Therefore, the sensitivity of the imprinted sensor towards AFPwill also be influenced by the pH value. Figure 6 shows the dependence of the peak current variation on the pH value. With the pH value increasing from 5.0 to 9.0, the current response to AFP increases dramatically and gets a maximum value at the pH value of 7.4. By further increasing pH value from 7.4 to 9.0, the current response decreases significantly. At the pH value of 7.4, AFP can maintain its configuration and charge property as it was used for the imprinted sensor fabrication, thus can be rebound to the imprinted sites efficiently.

#### 3.2.2. Effect of the Incubation Time

**Figure 7:**
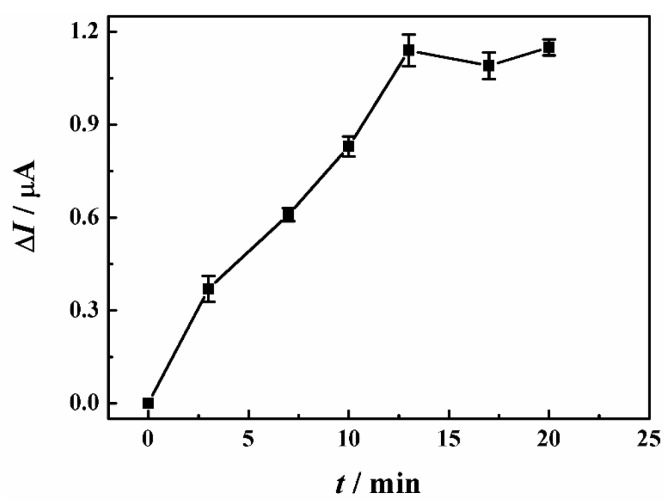
Influence of the incubation time on the current response of the imprinted sensor towards AFP at the concentration of 1.0 ng mL^−1^.

The incubation time is a critical parameter for evaluating the sensing performances of the imprinted sensor due that it can influence the rebinding amount of AFP to the imprinted film surface. The dependence of the current response variation on the incubation time was presented in Figure 7. It is found that the current response of the imprinted sensor towards AFP increases significantly with prolonging the incubation time from 0 to 12 min. A maximal value can be obtained at the incubation time of 12 min. By further increasing the incubation time, the current response decreased inversely, meaning nearly all imprinted sites have been occupied by AFP when being incubated in 1.0 ng mL^−1^ AFP solution for 12 min. Therefore, 12 min was selected as the optimal incubation time for AFP assay based on this imprinted sensor.

### 3.3. Analytical Characteristics

**Figure 8:**
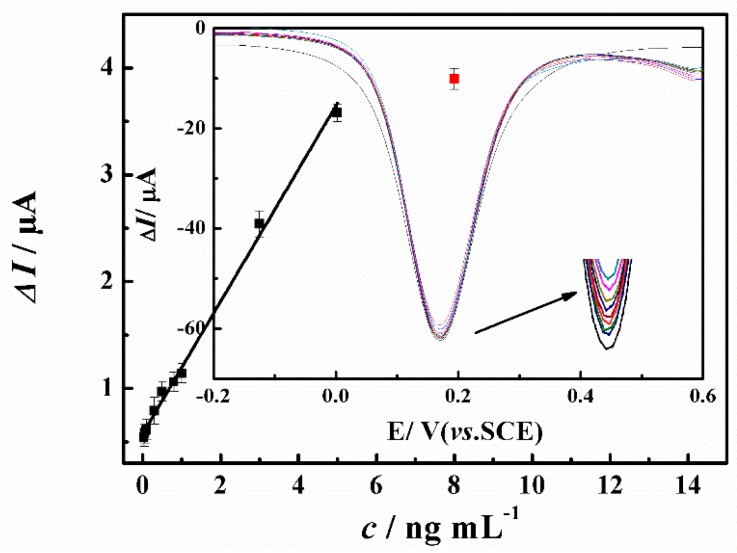
Calibration curve for determining AFP at the imprinted sensor. The inset is the differential pulse voltammograms of K_4_Fe(CN)_6_/K_3_Fe(CN)_6_ at the imprinted sensor with different AFP concentrations (0.03, 0.05, 0.1, 0.3, 0.5, 0.8, 1, 3, 5 ng mL^−1^).

Under optimized experimental conditions, the analytical characteristics of the imprinted sensor for determining AFP were evaluated. As shown in Figure 8, the current response of the imprinted sensor shows a linear dependence on the AFP concentration in the range from 0.03 ng mL^−1^ to 5 ng mL^−1^. The linear relationship can be depicted by the following equation: Δ*I* (μA) = 0.6186 *c* (ng mL^−1^) + 0.5778 (R = 0.992). The detection limit for AFP determination is calculated to be 0.002 ng mL^−1^ (S/N = 3). The DPV behavior of K_4_Fe(CN)_6_/K_3_Fe(CN)_6_ at the non-imprinted (NIP) sensor was also investigated. As shown in Figure S4, with AFP concentration varied from 0.03 to 1.0 ng mL^−1^, the peak current variation can be neglected, indicating no linear response towards AFP.

### 3.4. Selectivity of the Imprinted Sensor

**Figure 9:**
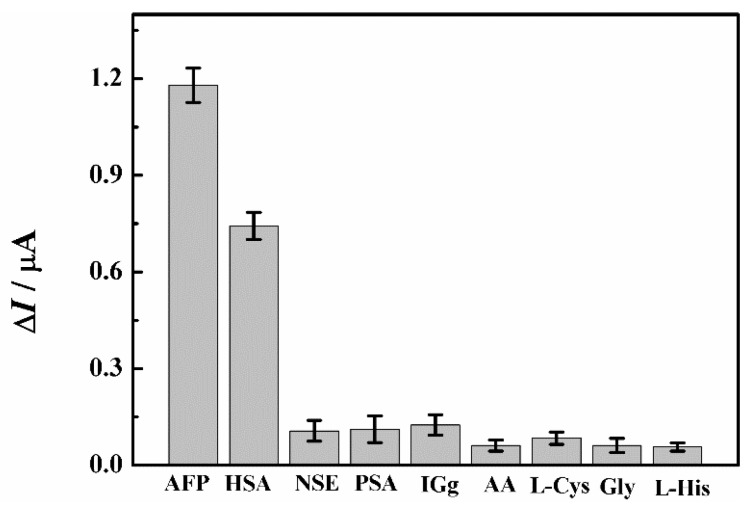
Current response of the imprinted sensor towards potential interferents.

**Figure 10:**
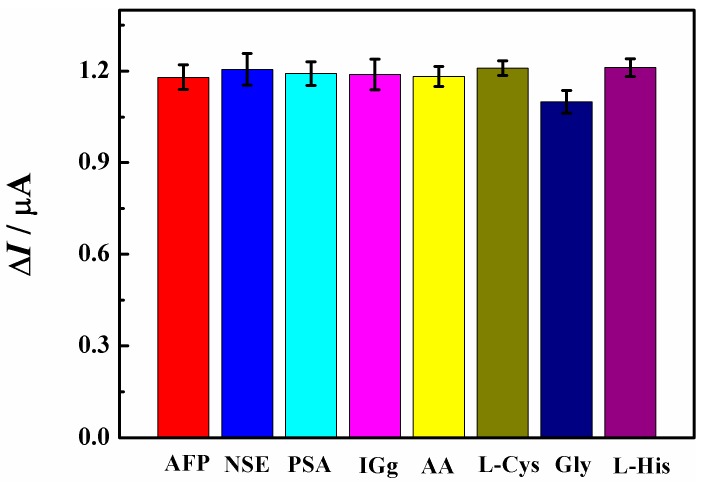
Electrochemical responses of the imprinted sensor toward 1.0 ng mL^−1^ AFP in the presence of 50 ng mL^−1^ NSE, PSA, IGg, AA, L-Cys, Gly and L-His.

The selective response towards AFP can be used to evaluate the imprinted efficiency. For the selectivity test, some proteins such as HSA, NSE, PSA, IgG and some bioactive molecules including AA, L-Cys, Gly, L-His were employed as potential interferents to investigate the selective sensing performance towards AFP. The concentrations of all molecules were controlled at 1.0 ng mL^−1^. Seen from Figure 9, the current response of AFP is much higher than others, meaning good selectivity towards AFP. Nevertheless, the response towards HSA is more sensitive than other protein and bioactive molecules due to possessing similar structure to that of AFP. Further experiments were carried out to test the selectivity of the AFP imprinted sensor. The electrochemical responses of the imprinted sensor toward 1.0 ng mL^−1^ AFP in the presence of 50 ng mL^−1^ NSE, PSA, IGg, AA, L-Cys, L-His, and Gly are shown in Figure 10, and it shows neglected variation, demonstrating high selectivity.

### 3.5. Reproducibility and Stability

Five imprinted sensors were fabricated independently and used for determining AFP at the concentration of 1 ng mL^−1^, respectively. The relative standard deviation (RSD) for the current response of AFP at these imprinted sensors is 4.52%, meaning excellent reproducibility for the imprinted sensor fabrication. In addition, one of the five imprinted sensors was selected for the successive determination of AFP at the concentration of 1 ng mL^−1^ for five times. A RSD of 4.81% indicates the imprinted sensor is suitable for determining AFP repetitively. To investigate the stability of the imprinted sensor, it was stored at ~4 °C for one week, then, was used to determine 1.0 ng mL^−1^ AFP again. Only a 7.41% decrease was observed for the peak current response compared with the initial response before being stored. The result demonstrated the imprinted sensor possesses good stability.

## 4. Conclusions

Gold nanoparticles were electrochemically deposited onto a glassy carbon electrode surface to develop a platform for fabricating an AFP imprinted sensor. By using 1-[3-(N-Cystamine)propyl]-3-vinylimidazolium tetrafluoroborate ionic liquid as functional monomer and AFP as template, a molecularly imprinted film was prepared by initializing the polymerization of the system at room temperature, thus, to help to maintain the bioactivity and configuration of AFP and to improve the selectivity of the imprinted sensor. All experimental results indicate an imprinted sensor was successfully fabricated with good selectivity, sensitivity, reproducibility and stability. This procedure will be suitable for preparing imprinted sensors for other biomolecules by substituting the template with corresponding molecules.

## Supplementary Materials

The following are available online at https://www.mdpi.com/1424-8220/19/14/3218/s1.

## References

[1] Wright L.M., Kreikemeier J.T., Fimmel C.J. (2007). A concise review of serum markers for hepatocellular cancer. Cancer Detect. Prev..

[2] Fang X., Li X.Q., Wang H., Wu X.M., Wang G.L. (2018). Tuning surface states to achieve the modulated fluorescence of carbon dots for probing the activity of alkaline phosphatase and immunoassay of alpha-fetoprotein. Sens. Actuators B Chem..

[3] Niu Y.L., Yang T., Ma S.S., Peng F., Yi M.H., Wan M.M., Mao C., Shen J. (2017). Label-free immunosensor based on hyperbranched polyester for specific detection of alpha-fetoprotein. Biosens. Bioelectron..

[4] Fan F., Shen H.Y., Zhang G.J., Jiang X.Y., Kang X.X. (2014). Chemiluminescence immunoassay based on microfluidic chips for α-fetoprotein. Clin. Chim. Acta.

[5] Zhang A.Y., Skog S., Wang S.Q., Ke Y., Zhang Y.H., Li K., He E., Li N. (2016). A Chemiluminescent protein microarray method for determining the seroglycoid fucosylation index. Sci. Rep..

[6] Liang X.L., Bao N., Luo X.L., Ding S.N. (2018). CdZnTeS quantum dots based electrochemiluminescent image immunoanalysis. Biosens. Bioelectron..

[7] Zheng X.L., Hua X.X., Qiao X.Y., Xia F.Q., Tian D., Zhou C.L. (2016). Simple and signal-off electrochemiluminescence immunosensor for alpha fetoprotein based on gold nanoparticle-modified graphite-like carbon nitride nanosheet nanohybrids. RSC Adv..

[8] Preechakasedkit P., Siangproh W., Khongchareonporn N., Ngamrojanavanich N., Chailapakul O. (2018). Development of an automated wax-printed paper-based lateral flow device for alpha-fetoprotein enzyme-linked immunosorbent assay. Biosens. Bioelectron..

[9] Wangkam T., Boonperm K., Khomkrachang P., Srikhirin T., Praphanphoj V., Sutapan B., Somboonkaew A., Amarit R. (2016). Hepatocellular carcinoma biomarker detection by surface plasmon resonance sensor. Adv. Mater. Res..

[10] Yang S.H., Zhang F.F., Wang Z.H., Liang Q.L. (2018). A graphene oxide-based label-free electrochemical aptasensor for the detection of alpha-fetoprotein. Biosens. Bioelectron..

[11] Wang Y.Y., Qu Y., Ye X.X., Wu K.B., Li C.Y. (2016). Fabrication of an electrochemical immunosensor for alpha-fetoprotein based on a poly-L-lysine-single-walled carbon nanotubes/prussian blue composite film interface. J. Solid State Electrochem..

[12] Zhou Q., Xue H.J., Zhang Y.Y., Lv Y.Q., Li H.G., Liu S.Q., Shen Y.F., Zhang Y.J. (2018). Metal-free all-carbon nanohybrid for ultrasensitive photoelectrochemical immunosensing of alpha-fetoprotein. ACS Sens..

[13] Chen X., Xu W., Jiang Y.D., Pan G.C., Zhou D.L., Zhu J.Y., Wang H., Chen C., Li D.Y., Song H.W. (2017). A novel upconversion luminescence derived photoelectrochemical immunoassay: Ultrasensitive detection to alpha-fetoprotein. Nanoscale.

[14] Lu C.X., Tang Z.G., Guo X.X., Ma X.M., Liu C.B. (2018). Computer-aided design of magnetic dummy molecularly imprinted polymers for solid-phase extraction of ten phthalates from food prior to their determination by GC-MS/MS. Microchim. Acta.

[15] Rutkowska M., Plotka-Wasylka J., Morrison C., Wieczorek P.P., Namiesnik J., Marc M. (2018). Application of molecularly imprinted polymers in analytical chiral separations and analysis. TrAC Trends Anal. Chem..

[16] Mirata F., Resmini M. (2015). Molecularly Imprinted Polymers in Biotechnology. Molecularly Imprinted Polymers for Catalysis and Synthesis.

[17] Zhang X., Yang S., Jiang R., Sun L.Q., Pang S.P., Luo A.Q. (2018). Fluorescent molecularly imprinted membranes as biosensor for the detection of target protein. Sens. Actuators B Chem..

[18] Wang X., Wang Y.Y., Ye X.X., Wu T.S., Deng H.P., Wu P., Li C.Y. (2018). Sensing platform for neuron specific enolase based on molecularly imprinted polymerized ionic liquids in between gold nanoarrays. Biosens. Bioelectron..

[19] Wang C.Y., Ye X.X., Wang Z.G., Wu T.S., Wang Y.Y., Li C.Y. (2017). Molecularly imprinted photo-electrochemical sensor for human epididymis protein 4 based on polymerized ionic liquid hydrogel and gold nanoparticle/ZnCdHgSe quantum dots composite film. Anal. Chem..

[20] Yanez-Sedeno P., Campuzano S., Pingarron J.M. (2017). Electrochemical sensors based on magnetic molecularly imprinted polymers: A review. Anal. Chim. Acta.

[21] Mo G.C., He X.X., Zhou C.Q., Ya D.M., Feng J.S., Yu C.H., Deng B.Y. (2019). A novel ECL sensor based on a boronate affinity molecular imprinting technique and functionalized SiO_2_@CQDs/AuNPs/MPBA nanocomposites for sensitive determination of alpha-fetoprotein. Biosens. Bioelectron..

[22] Lai Y.X., Zhang C.X., Deng Y., Yang G.J., Li S., Tang C.L., He N.Y. (2019). A novel alpha-fetoprotein-MIP immunosensor based on AuNPs/PTh modified glass carbon electrode. Chin. Chem. Lett..

[23] Shen X.L., Ma Y., Zeng Q., Tao J., Wang L.S. (2016). Molecularly imprinted electrochemical sensor for advanced diagnosis of alpha-fetoprotein. Anal. Methods.

[24] Wang Y.Y., Han M., Ye X.X., Wu K.B., Wu T.S., Li C.Y. (2017). Voltammetric myoglobin sensor based on a glassy carbon electrode modified with a composite film consisting of carbon nanotubes and a molecularly imprinted polymerized ionic liquid. Microchim. Acta.

[25] Wang Y.Y., Han M., Liu G.S., Hou X.D., Huang Y.N., Wu K.B., Li C.Y. (2015). Molecularly imprinted electrochemical sensing interface based on in-situ-polymerization of amino-functionalized ionic liquid for specific recognition of bovine serum albumin. Biosens. Bioelectron..

[26] Zhao B.T., Li C.Y. (2015). Synthesis and characterization of 1-(3-(N-Cysimine) propyl)-3-methylimidazolium bromine ionic liquid. GuangZhou Chem. Ind..

