# Supplementary material for: Electrochemical Sensing of α-Fetoprotein Based on Molecularly Imprinted Polymerized Ionic Liquid Film on a Gold Nanoparticle Modified Electrode Surface
Source: Sensors (Basel). 2019 Jul 22;19(14):3218. doi: 10.3390/s19143218 (PMC6679504; doi:10.3390/s19143218)
Supplement: Supplementary file 1 [file sensors-19-03218-s001.pdf]

High-performance platform for electrochemical sensing α-fetoprotein based on molecularly imprinted polymerized ionic liquid film on a gold nanoparticle modified electrode surface

Yingying Wu[b,1], Yanying Wang[b,1], Xing Wang[b], Chen Wang[b], Chunya Li[b,*], Zhengguo Wang[a,*]

[a]*Institute of Food Science and Engineering Technology, Hezhou University, Hezhou, Guangxi 542899, China*

[b]*Key Laboratory of Analytical Chemistry of the State Ethnic Affairs Commission, College of Chemistry and Materials Science, South-Central University for Nationalities, Wuhan 430074, China*

* Corresponding Author.

E-mail: lichychem@163.com & wangzghz@sina.com.

[1]These authors contributed equally to this work.

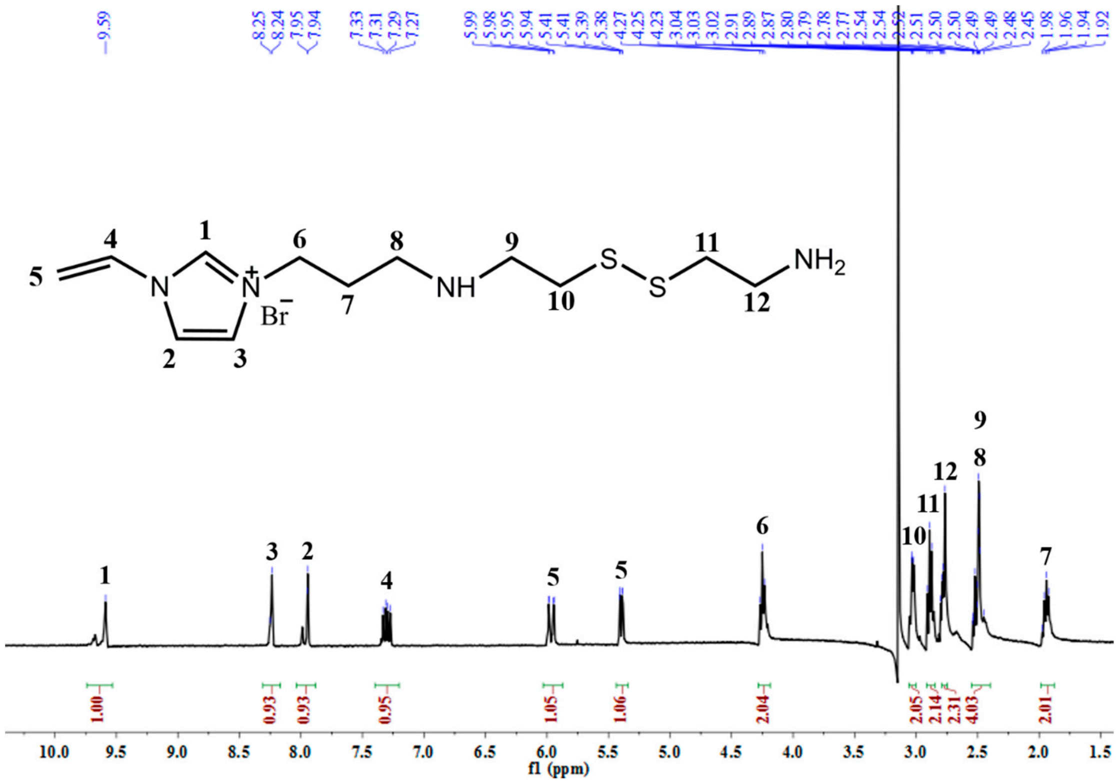

Fig. S 1. ^{1}H-NMR spectrum of (Cys)VIMBF$_4$ ionic liquid (solvent: Methanol-d_4)

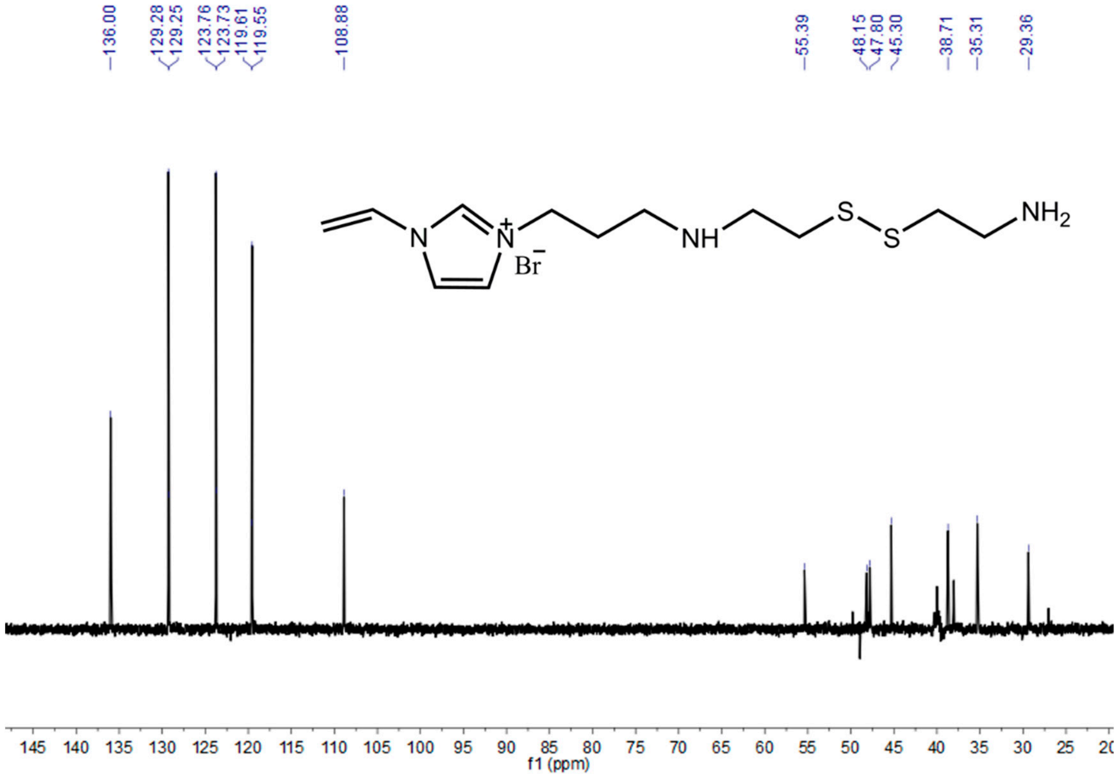

Fig. S 2. ^{13}C-NMR spectrum of (Cys)VIMBF$_4$ ionic liquid (solvent: Methanol-d_4)

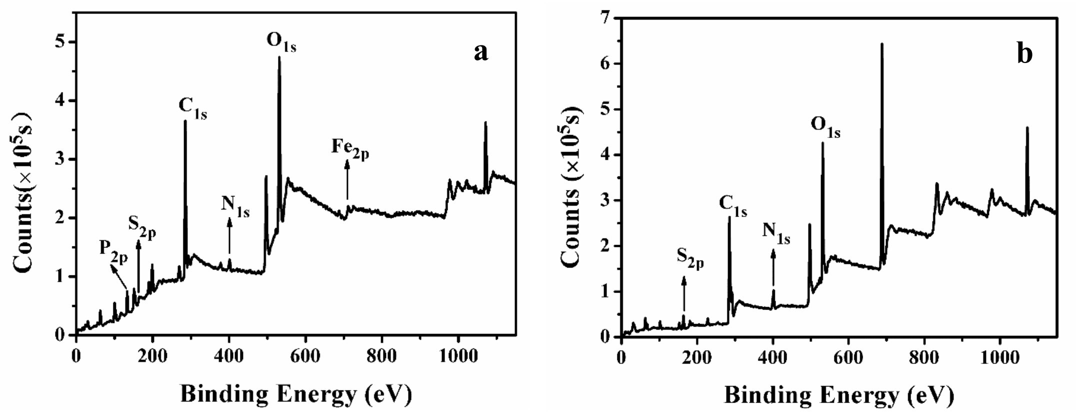

Fig. S3. X-ray photoelectron spectroscopic characterizations of imprinted (a) and non-imprinted polymers (b)

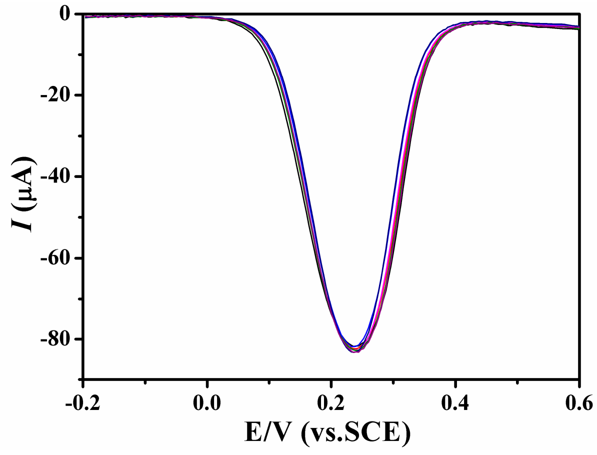

Fig. S 4 Differential pulse voltammograms of $K_4Fe(CN)_6/K_3Fe(CN)_6$ at the NIP sensor with AFP concentration varying from 0.03 to 1.0 ng mL^{-1}

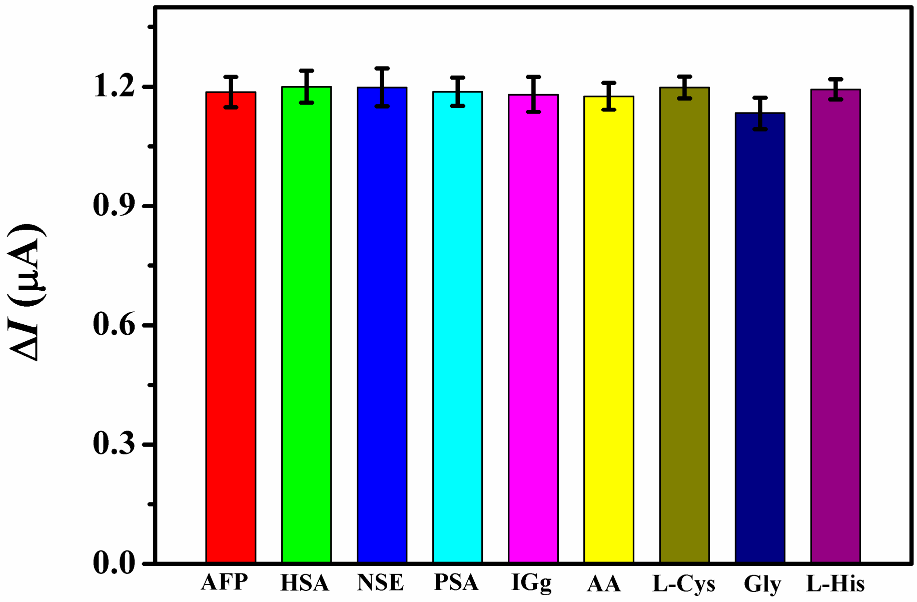

Fig. S 5. Electrochemical responses of the imprinted sensor toward 1.0 ng mL^{-1} AFP in the presence of 50 ng mL^{-1} HSA, NSE, PSA, IGg, L-Cys, Gly and L-His.
